# Cancer Screening in Patients with Unprovoked Thromboembolism: How to do it and Who Benefits?

**DOI:** 10.7759/cureus.6934

**Published:** 2020-02-10

**Authors:** Filipa Ferreira, José Pereira, Ana Lynce, José Nunes Marques, Ana Martins

**Affiliations:** 1 Medical Oncology, Centro Hospitalar de Lisboa Ocidental, Lisbon, PRT; 2 Internal Medicine, Centro Hospitalar de Lisboa Ocidental, Lisbon, PRT

**Keywords:** unprovoked thromboembolism, cancer screening, thromboembolism, cancer, mortality

## Abstract

Introduction: Unprovoked venous thromboembolism (uVTE) may be the first manifestation of cancer. The main objectives of this study were to compare limited screening (LS) and extended screening (ES) and to make a protocol to approach these patients.

Methods: This is a retrospective, unicentric observational study that included 245 patients with venous thromboembolism (VTE) admitted to an Internal Medicine Service for five years. The incidence of cancer and mortality during hospitalization, and at one and three years after admission were calculated in both LS and ES groups and compared.

Results: Of the 245 patients with VTE, 59 (24.1%) had uVTE: 35 (59.3%) were submitted to LS and 24 (40.7%) to ES, with 10 (4.1%) diagnosis of cancer. In the following three years, 10 more patients were diagnosed. There were no statistically significant differences in inpatient diagnosis rates (8.6% vs. 4.2%; p=0.51) or in-hospital mortality (2.9% vs. 4.2%; p=0.79) or mortality at one year (8.6% vs. 8.3%; p=0.97) and three years (20.0% vs. 20.8%; p = 0.94) between LS and ES groups respectively. The Computerized Registry of Patients with Venous Thromboembolism (RIETE) score was equal or superior to 3 in 69.5% (N=41) of the population with uVTE.

Discussion: The results of our study are consistent with the literature; there are no differences between screenings, as the difference in the number of diagnoses does not reflect on mortality.

Conclusion: There were no statistically significant differences between the two types of screening in this population. We suggest a protocol that includes the RIETE score to better select the patients who might benefit the most from an ES.

## Introduction

Cancer is a well-established independent risk factor for venous thromboembolism (VTE), and an episode of unprovoked VTE (uVTE) may be the first manifestation of a hidden neoplasm [1-3]. The incidence of cancer associated with uVTE is about 4.5%-5%, with the risk being particularly high in the first six months after the episode and higher in colorectal, pancreatic, brain and lung tumours [3-6]. Most studies show a risk reduction after the first 12 months, equalling the general population, but others show increased rates up to six years [4,5,7].

Cancer screening after an episode of uVTE may allow an early diagnosis of occult cancer. However, the investigation of patients with uVTE lacks national or international guidelines that clarify what types of tests should be done and in which patients. Several trials compare two types of screening: limited and extended. Limited screening (LS) strategies are variable in the literature, but usually include clinical history, physical examination, general analyses (blood count, kidney function, ionogram, and liver function) and chest X-ray; some trials also include screening tests related to the patient's age and gender [1,5]. Besides limited screening examinations, extended screening (ES) includes additional tests such as ultrasound, computed tomography (CT) or positron emission tomography/CT (PET/CT) [5]. Most trials conclude that LS diagnoses up to about 90% of occult neoplasms, with a difference in the number of diagnoses between the two types of screening that is not reflected in mortality [5,8-12]. ES is also associated with more false positives, unnecessary research and higher costs [5,12]. The 2012 National Institute for Health Care Excellence (NICE) guidelines suggest that all patients should undergo medical history, physical examination, general analysis, and chest X-ray, and that abdominal-pelvic CT should be performed on patients older than 40 years and mammography in all women [5]. On the other hand, the 2016 guidelines of the Anticoagulation Forum suggest that patients should undergo LS, with clinical history, physical examination, general analysis, chest X-ray and age/gender-appropriate screening [13].

Several clinical and analytical factors have revealed a predictive value for the identification of patients with uVTE at increased risk of cancer [14-17]. The Computerized Registry of Patients with Venous Thromboembolism (RIETE) score was validated for this purpose, with the highest risk being present in patients who meet three or more of the following criteria: male gender, age equal or superior to 70 years, chronic lung disease, anemia, thrombocytosis, previous VTE and recent surgery [18]. However, it remains unclear which patients should undergo more extensive screening and whether the earlier detection of cancer improves patients' morbidity, mortality, and quality of life.

The main objectives of this study are: to compare the two types of screening (LS and ES) through the evaluation of the exams performed on the population with uVTE in an Internal Medicine Service over five years, the calculation of the incidence of cancer (during hospitalization, in the first year and in the second and third years after) and evaluation of its impact on mortality, and depending on the data obtained, to develop a protocol to approach these patients. As secondary objectives, it is also intended to evaluate the characteristics of the various patient subgroups and calculate the RIETE score for the study population.

## Materials and methods

Study design

This is an observational, retrospective, and single-centre study, which includes patients hospitalized with the diagnosis of VTE in the Internal Medicine Service of Hospital São Francisco Xavier over a period of five years, between January 1, 2011 and December 31, 2015. Patients older than 18 years and diagnosed with VTE according to ICD-9 (International Statistical Classification of Diseases and Related Health Problems 9) were included. The selection of patients was performed using the Homogeneous Diagnostic Groups, a Portuguese classification system of the diagnoses of hospitalized patients in acute hospitals. The following diagnoses and respective codes were considered: 415.1 - Embolism and pulmonary infarction, 415.19 - Pulmonary thromboembolism (PTE), 453 - Embolism and venous thrombosis, not elsewhere classified, 453.2 - Embolism and thrombosis of the vena cava, 453.3 - Embolism and thrombosis of the renal vein, 453.4 - Embolism and venous thrombosis of unspecified deep vessels of the limbs, 453.5 - Embolism and chronic thrombosis of deep lower extremity vessels, 453.6 - Embolism and thrombus of chronic of superficial vessels of lower extremity, 453.7 - Embolism and chronic thrombosis of specified vessels not classified elsewhere, 453.8 - Embolism and thrombosis of specified veins not elsewhere classified and 453.9 - Embolism of thrombosis of unspecified location.

uVTE was defined as the VTE that occurs in the absence of a previous history of VTE, active neoplasia, coagulopathy, hormonal therapy, pregnancy/puerperium, or surgery, trauma or immobilization in the previous three months. LS was defined as the screening that includes clinical history, physical examination, general analyses (blood count, renal function, ionogram, and liver function), chest X-ray (and/or CT angiography of the chest in case of PTE) and screening appropriate to age and gender (breast examination and/or mammography in women older than 50 years, colpocytology in sexually active women aged between 18 to 70 years; rectal examination and/or prostate specific antigen (PSA) in men older than 40 years; colonoscopy in patients older than 50 years). ES included, besides LS tests, others such as analytic panel of tumour markers, autoimmune analytic profile, analytic study of thrombophilia, ultrasound, CT, nuclear magnetic resonance (NMR), endoscopic exams or other invasive examinations.

Data collection was carried out by consulting the hospital clinical process through the Portuguese platform SAM (Sistema de Apoio ao Médico - Physician Support System) and extra-hospital through the Portuguese RSE (Registo de Saúde Eletrónico - Electronic Health Record). The variables collected were: gender; birth date; characteristics of the thromboembolic phenomenon (PTE and/or deep vein thrombosis (DVT)); comorbidities, including the existence of previous VTE and a known neoplasm (as well as its characteristics - primary tumour, stage, active treatment); diagnosis of cancer during hospitalization (tests performed, primary tumour, stage); anticoagulant therapy (inpatient and outpatient); diagnosis of cancer in the following year and 2-3 years thereafter (primary tumour, stage); mortality of patients diagnosed with cancer (inpatient and after discharge).

Statistical analysis

Variables of a continuous nature are presented as mean (± standard deviation) or median (interquartile range), depending on the underlying distribution of the data. Variables of a discrete nature are presented as absolute and relative frequencies. The Chi-square test was used to compare categorical variables and the Student's t-test was used to compare continuous variables. Values ​​of p <0.05 were considered statistically significant. The data were analysed using Microsoft Excel and Statistical Package for the Social Sciences (SPSS Inc., Chicago, IL), version 25.

## Results

During the period considered, 4538 patients were admitted to the Internal Medicine Service, of which 245 (5.40%) were diagnosed with VTE. The median age of the patients was 75 years and 88 patients (35.9%) were male. Regarding the embolic phenomenon, most patients (N=138; 56.3%) had a diagnosis of PTE, 60 (24.5%) had DVT and 47 (19.2%) had both. Among comorbidities, 62.9% (N=154) patients had arterial hypertension, 31.4% (N=77) had dyslipidaemia, 22.0% (N=54) had diabetes, 12.7% (N=31) had a previous episode of VTE (13 of these still under therapeutic anticoagulation) and 25.7% (N=63) had a previous diagnosis of cancer. Of the patients with known cancer, the majority had gastrointestinal (31.7%; N=20), genitourinary (22.2%, N=14) or breast (14.3%; N=9) tumours, with 41.3% in stage IV and 65.1% with no active treatment at the time of admission. The characteristics of the general population can be seen in Table 1 and those of the subgroup with known diagnosis of cancer in Table 2.

**Table 1:** Characteristics of the general population VTE: venous thromboembolism.

General Population (N=245)		
Gender (N; %)	Male	88 (35.9%)
	Female	157 (51.8%)
Median age (years) [Min;Max]		75 [18;98]
VTE (N; %)	Pulmonary thromboembolism	138 (56.3%)
	Deep vein thrombosis	60 (24.5%)
	Both	47 (19.2%)
Comorbidities (N; %)	Arterial hypertension	154 (62.9%)
	Diabetes	54 (22.0%)
	Chronic lung disease	24 (9.8%)
	Coronary disease	14 (5.7%)
	Dyslipidaemia	77 (31.4%)
	Obesity	35 (14.3%)
	Coagulopaties	5 (2.0%)
	Cancer	63 (25.7%)
	Tabagism	34 (13.9%)
	Alcoolism	13 (4.3%)
	Surgery, trauma or immobilization in the last 3 months	29 (11.8%)
	Pregnancy or puerperium	2 (0.8%)
	Hormonal therapy	2 (0.8%)
Previous VTE (N; %)	Total	31 (12.7%)
	< 6 months	9 (3.7%)
	≥ 6 months	12 (4.9%)
	Unknown	10 (4.1%)
	Under anticoagulation	13 (4.3%)
Anticoagulation (N; %)	Inpatient	
	Low-molecular-weight heparin	196 (80.0%)
	Low-molecular-weight heparin with bridge to Warfarin	31 (12.7%)
	Low-molecular-weight heparin with bridge to Direct Oral Anticoagulants	8 (3.3%)
	Thrombolysis followed by Low-molecular-weight heparin	10 (4.1%)
	Outpatient	
	Low-molecular-weight heparin	62 (25.3%)
	Warfarin	97 (39.6%)
	Direct Oral Anticoagulants	52 (21.2%)
	Not instituted for clinical reasons	14 (5.7%)
Mortality during hospitalization (N; %)	Total	20 (8.2%)
	Related to known cancer	9 (3.7%)
	Related to inpatient cancer diagnosis	2 (0.8%)
	Other causes	9 (3.7%)
VTE recurrence	Total	18 (7.3%)
	Cancer patients	10 (4.1%)

**Table 2:** Characteristics of the group of patients with known cancer at admission

Patients with known cancer at admission (N=63)		
Primary tumour (N; %)	Gastrointestinal	20 (31.7%)
	Urological	14 (22.2%)
	Breast	9 (14.3%)
	Hematologic	6 (9.5%)
	Gynecological	4 (6.3%)
	Thoracic	3 (4.8%)
	Occult primary	3 (4.8%)
	Central Nervous System	3 (4.8%)
	Soft tissues	1 (1.6%)
Stage (N; %)	I-III	35 (55.6%)
	IV	26 (41.3%)
	Unknown	2 (3.2%)
Treatment (N; %)	No active treatment	41 (65.1%)
	Under active treatment	22 (34.9%)
	Chemotherapy	14 (22.2%)
	Hormonal therapy	8 (12.7%)
	Central venous catheter	12 (19.0%)

**Figure 1:**
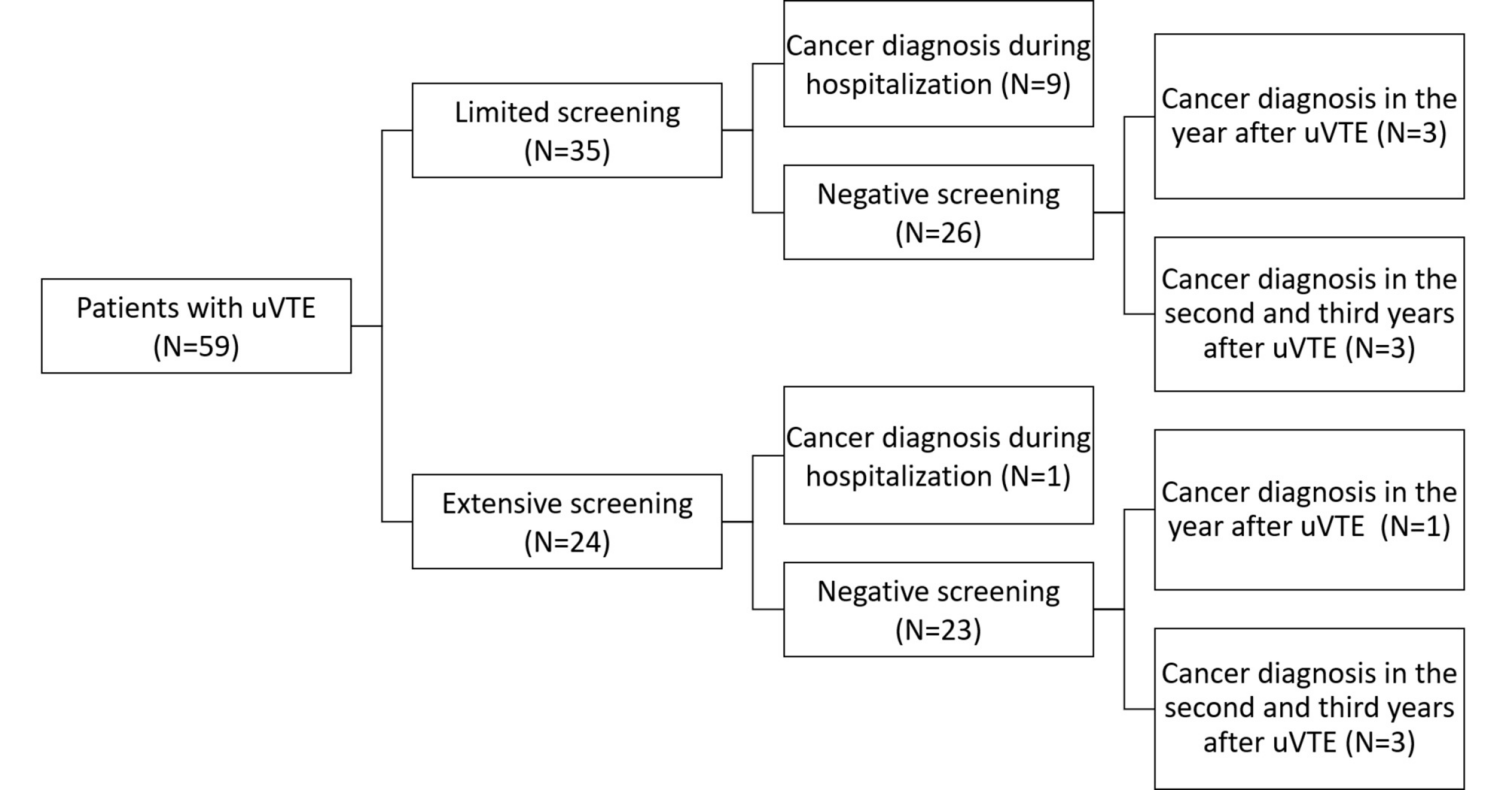
Scheme illustrating the results of screening for patients with unprovoked venous thromboembolism (uVTE)

Analysing the personal history of the patients, accordingly with the definition of uVTE, 186 patients were excluded, since they presented risk factors for provoked VTE. The remaining 59 (24.1%) did not have any evident risk factors for VTE, whereby they had an episode of uVTE. Of these, 35 (59.3%) underwent limited screening tests and 24 (40.7%) underwent ES (Table 3) (Figure 1).

**Table 3:** Tests performed on patients with unprovoked venous thromboembolism (uVTE)

Tests performed on patients with uVTE (N=59)	
Clinical history and physical examination	59 (100%)
General analysis	59 (100%)
Panel of tumor markers	15 (25.4%)
Autoimmune profile and study of thrombophilia	8 (13.6%)
Chest X-Ray (and/or thoracic AngioTC if PTE)	59 (100%)
Screening tests suitable for age and gender	6 (10.2%)
Ultrasound	31 (52.5%)
Computed tomography and nuclear magnetic resonance	7 (11.9%)
Endoscopic exams	3 (5.1%)

There were ten inpatient diagnoses of cancer, nine of which in the subgroup that underwent LS. In the year following admission, four patients were diagnosed with cancer during the follow-up by their attending physicians: one patient had undergone ES and the remaining three LS. In the second and third year after hospitalization, six more patients were diagnosed, three in each group. In both groups, 60.0% of patients had cancer at an advanced stage at time of diagnosis. These data can be seen in Table 4 and Figure 1.

**Table 4:** Characteristics of patients with cancer diagnosed during or after hospitalization

Patients with cancer diagnosed during or after hospitalization (N=20)					
		Limited screening		Extended screening	
		Localized disease	Advanced disease	Localized disease	Advanced disease
During hospitalization (N=10)	Urological	0	1	0	0
	Gynecological	0	1	0	0
	Hematologic	0	2	0	0
	Thoracic	0	2	0	0
	Central nervous system	1	0	0	0
	Endocrine	0	0	1	0
	Occult primary	0	2	0	0
First year after hospitalization (N=4)	Gastrointestinal	2	0	0	1
	Central nervous system	1	0	0	0
Second and third years after hospitalization (N=6)	Gastrointestinal	1	1	1	0
	Gynecological	0	0	0	1
	Hematologic	1	0	0	0
	Occult primary	0	0	0	1
Total		6	9	2	3
		15		5	

Calculating the RIETE score for the population with uVTE, it is concluded that 41 patients had a score equal or above 3, of which 28 underwent ES. This subgroup included five patients who were diagnosed with cancer during hospitalization (only one who underwent ES) and four of those who were diagnosed in the following years and had undergone LS.

The rate of VTE recurrence was 7.3% (N=18), with more than half of the patients (N=10; 4.1%) presenting a diagnosis of cancer, already known or diagnosed during or after hospitalization. In-hospital mortality was 8.2%, with eleven patients dying from cancer, two of whom were diagnosed during hospitalization (one from each screening group). There was no statistically significant difference in in-hospital mortality between the LS and ES groups: 2.9% vs. 4.2% (p=0.79), respectively. Of the remaining eight patients diagnosed on admission, three died in the first year and two in the following 2-3 years. Of the ten patients diagnosed after hospitalization, six died in the following two years. There were also no statistically significant differences between mortality rates at one year (8.6% vs. 8.3%; p=0.97) and at three years (20.0% vs. 20.8%; p=0.94) between the LS and ES groups respectively.

## Discussion

An occult cancer is detected in 4.5%-5.0% of patients with VTE and several studies show that LS diagnoses up to 90% of hidden neoplasms in patients with uVTE [5,6,11,19]. It was part of the main objective of this study to calculate the incidence rate of cancer diagnosis in hospitalized patients with VTE, which was 10 out of 245 (4.1%) of the patients. In the inpatient regime, limited screening diagnosed 90% of the neoplasms (9 out of 10).

Screening techniques differ between trials, both in relation to LS, which only in some trials include screening tests related to patients age and gender, and in relation to ES, in which most trials include CT or PET/CT but others also include endoscopic exams. This fact makes it difficult to compare the data, but the conclusions are generally coincident. The Subsequent Diagnosis Of Malignancy in Patients presenting with Idiopathic Venous Thromboembolism (SOMIT) trial in 2004 showed that the difference in diagnoses between the two screening approaches was not statistically significant (10% in LS and 14% in ES) and that, although the diagnoses were made earlier in the ES group, this difference was not reflected in mortality [8,12]. Between 2011 and 2016, three trials were published comparing the two types of screening. The Trousseau trial compared LS with an ES including abdominal-pelvic CT and mammography, which was not associated with improved prognosis [12]. In the Screening for Occult Malignancy in Patients with Idiopathic Venous Thromboembolism (SOME) randomized trial, the number of cancer diagnoses did not increase by the addition of thoraco-abdominal-pelvic CT and tumour markers to the screening and there was no difference in time until diagnosis or mortality [9]. Similarly, Prandoni et al. concluded in a randomized, multicentre trial with 195 patients, that CT-based screening and faecal occult blood testing does not bring significant benefit over LS based on clinical criteria [10]. The Standard Diagnostic Procedures With or Without Fludeoxyglucose F 18 Positron Emission Tomography in Finding Cancer in Patients With a Blood Clot in a Vein (MVTEP) randomized trial is the largest to evaluate the inclusion of PET/CT in the screening, having shown that no more neoplasms were detected in a statistically significant percentage, but PET/CT had an important predictive value, with the risk of cancer being lower after negative PET/CT than after a negative LS [2,20,21]. A Cochrane review published in 2015 revealed that there was insufficient evidence that additional tests besides LS reduced morbidity or mortality [5,22]. A meta-analysis that included the 1,830 patients of Trousseau, SOME and MVTEP trials, concluded that ES was not effective in reducing mortality, which the authors associated with the reduced number of cancer diagnoses (N=98; 5.4%) and the heterogeneity of screening between tests and samples [6]. Another meta-analysis, by Zhou M. et al., with 2274 patients from five randomized trials, reached similar conclusions, with no difference in the risk of non-diagnosis or mortality between the two types of screening [11].

The main objective of our study was to compare the two types of screening by calculating the incidence of cancer (during hospitalization, in the first year and in the second and third years after) and assessing their impact on mortality. As this is a retrospective study and the tests performed depended on the decision of the attending medical team, the exams performed were not coincident among the various patients in each screening subgroup. However, the inclusion in the definition of LS or ES was similar with what was performed in most trials. There were no statistically significant differences between the rates of inpatient cancer diagnosis (8.6% vs. 4.2%; p = 0.51) or in mortality rates during hospitalization (2.9% vs. 4.2%; p = 0.79), at one year (8.6% vs. 8.3%; p = 0.97) and three years (20.0% vs. 20.8%; p = 0.94) between the LS and ES groups respectively. These results are compatible with other trials in which the median age and rate of detection of neoplasms in the advanced stage was similar to our analysis. Several trials concluded that patients diagnosed with cancer after uVTE had a poor prognosis, with most deaths occurring in the first year of follow-up. The authors of these trials raise the possibility that the association of VTE with cancer may reflect a more aggressive disease and which would probably become symptomatic soon, so an earlier diagnosis may not have an impact on the prognosis [6,12].

The ideal follow-up time after an episode of uVTE is not yet fully established. The risk of diagnosing cancer is highest in the first six months and remains high for at least twelve months, but some studies show that it remains higher than in the general population up to six years after uVTE [3-6]. For this reason, in our study, cancer diagnosis data were collected up to one year and up to the third year after the uVTE episode. There were four diagnoses in the first year and six in the second and third years, which corresponds respectively to 6.8 and 10.2% of the population with uVTE, in which 40.0% (N=4) had undergone ES. These data raise the possibility that the risk of cancer might indeed remain high for more than the first year after the episode, but it is not possible to draw conclusions.

The cost-effectiveness analysis of including CT in the screening of the Trousseau and SOME trials showed that it is not cost-effective, as it requires more costs without bringing greater efficacy in detecting occult cancer than LS [12,23]. The post-hoc analysis of the cost-benefit of adding PET/TC to screening in the MVTEP trial, however, showed that this may be a valid option to consider since, although more expensive, screening with PET/TC lead to less late diagnoses and improved quality of life when compared to LS [24]. Since the screenings were not uniform in our study, it was not possible to carry out this analysis.

When calculating the RIETE score for the population with uVTE, we concluded that 69.5% (N=41) had a score equal or above 3 and, therefore, a risk of occult cancer superior to 10%. According to the score validation trials, this may be the subgroup that benefits the most from ES [18]. In these trials, one-third of the population was at high risk, which leads to the conclusion that our population would have a higher risk. Of the 41 patients who had a score equal or above 3, only 28 underwent ES. In this group, were included 8 patients with a diagnosis of cancer during hospitalization or in the 1-3 years after who had undergone LS. These data raise the possibility that the approach to these patients could have been more appropriate if the RIETE score had been used to identify high-risk patients.

The main limitations of this study relate to the fact that it is a single-centre and retrospective study, which interferes in the study design and in the data collection, which was made based on computer records. The retrospective design makes it difficult to standardize the screenings in the study, as the exams were ordered by different medical teams at different times in the five-year period. The sample size is also small, which is an obstacle highlighted in several trials given the fact that only about 5% of patients with uVTE present a diagnosis of cancer.

**Figure 2:**
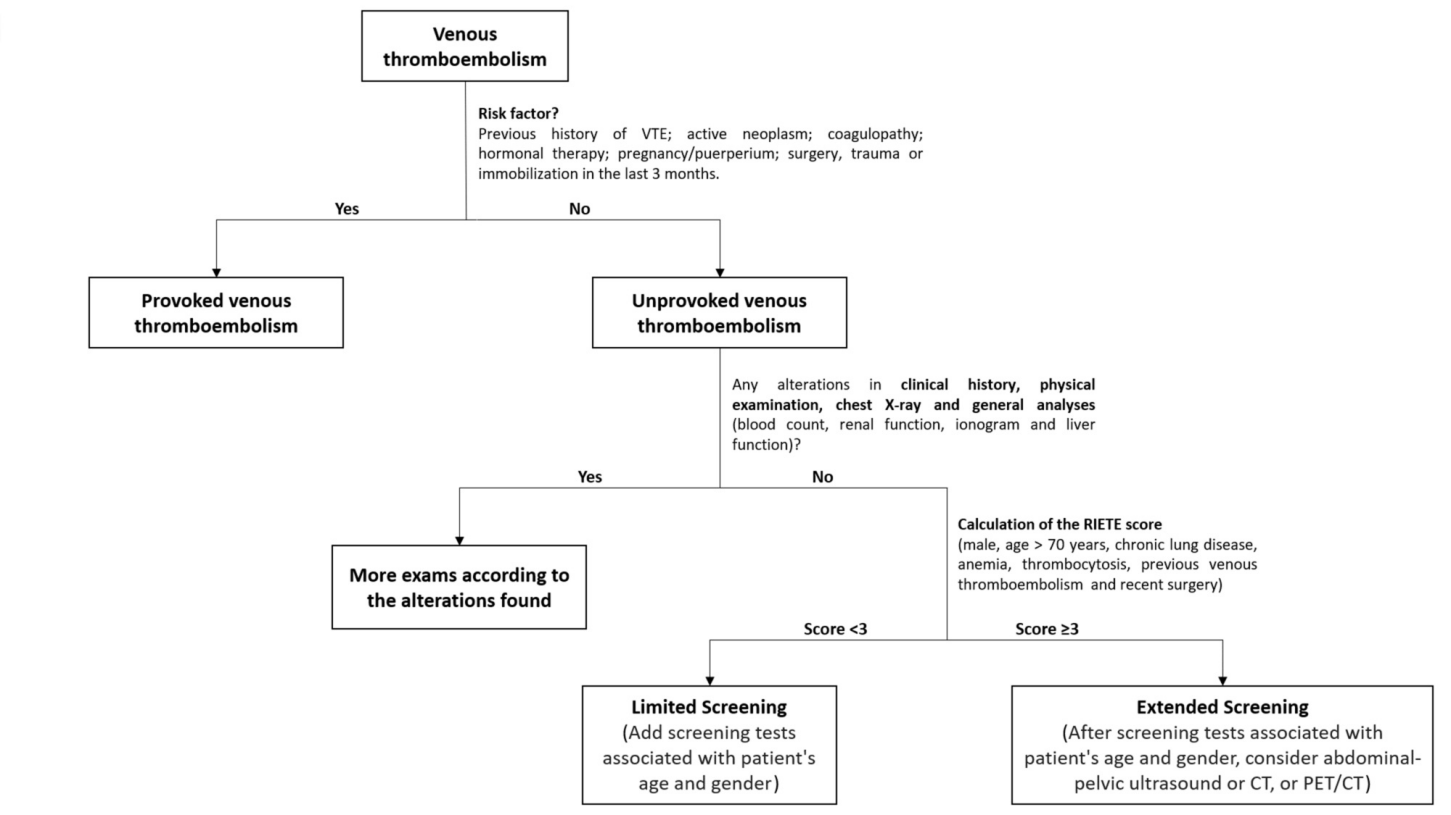
Protocol for the approach of patients with uVTE CT: computed tomography; PET: positron emission tomography.

A second point in the main objectives of this work was to develop a protocol for the approach of patients with uVTE. Thus, based on a literature review and this retrospective analysis, we suggest the protocol that can be observed in Figure 2. It is an objective that, depending on the approval of the protocol by the Internal Medicine Service of our hospital, it can be implemented, and a prospective study can be designed to analyse the results.

## Conclusions

In this study, the incidence of cancer diagnosis in patients with VTE was 4.1% and there were no statistically significant differences in the rates of inpatient cancer diagnosis (8.6% vs. 4.2%; p = 0.51) or in mortality rates during hospitalization (2.9% vs. 4.2%; p=0.79), at one year after (8.6% vs. 8.3%; p=0.97) and at three years after (20.0% vs. 20.8%; p=0.94) between the LS and ES groups respectively, with both results being similar to most of the literature. When calculating the RIETE score, which predictive value is validated for the population with uVTE, we concluded that 69.5% (N=41) had a score equal or above 3, which is equivalent to a risk of occult cancer superior to 10%. It is important that scores like this are included in clinical practice and that future studies focus on identifying subgroups that may benefit from more intensive screening strategies, in order to increase the number of diagnoses without increasing the costs and the number of futile procedures.
